# Comparing the psychometric properties of EQ-5D-3L and EQ-5D-5L proxy ratings by informal caregivers and a health professional for people with dementia

**DOI:** 10.1186/s12955-022-02049-y

**Published:** 2022-10-05

**Authors:** Bernhard Michalowsky, Wolfgang Hoffmann, Wiebke Mohr, Anika Rädke, Feng Xie

**Affiliations:** 1 German Center for Neurodegenerative Diseases (DZNE), Site Rostock/Greifswald, Ellernholzstraße 1-2, 17489 Greifswald, Germany; 2 Epidemiology of Health Care and Community Health, Institute for Community Medicine, University Medicine Greifswald, Greifswald, Germany; 3 Health Research Methods, Evidence and Impact, McMaster University, Hamilton, Canada; 4 Centre for Health Economics and Policy Analysis, McMaster University, Hamilton, Canada

**Keywords:** EQ-5D, Quality of life, Alzheimer's diseases, Dementia

## Abstract

**Background:**

Assessing health-related quality of life (HRQoL) among persons with dementia poses several challenges due to cognitive decline and limited perception. As a result, proxy ratings by family members or health professionals are used. The EQ-5D is the most commonly used generic and preference-based HRQoL instrument. Methodological drawbacks of the three-level version (EQ-5D-3L) prompted the development of the five-level version (EQ-5D-5L) by expanding the range in the domains. However, no comparison of the psychometric properties of both versions and different proxy ratings exist so far. Therefore, the objective of this study was to compare the psychometric properties of the EQ-5D-5L and EQ-5D-3L by application of different proxy ratings in dementia.

**Methods:**

The EQ-5D-3L and -5L were completed by n = 52 family caregivers and one care manager at baseline and three and six months later. In total, 106 caregiver and 133 care manager proxy ratings were completed. The EQ-5D-3L and 5L were evaluated in terms of acceptability (missing values), agreement, ceiling effects, redistribution properties and inconsistency, and informativity (Shannon, H', and Shannon Evenness, J', indices) as well as convergent and discriminative validity.

**Results:**

Mean proxy index scores were higher for the 5L than the 3L. Missing values occurred less frequently in both proxy ratings and versions (< 1%). Agreement between both measures was high but higher in caregiver than care-manager ratings (ICC 0.885 vs. 0.840). The relative ceiling effect decreased from the 3L to the 5L, more intensively in the care-manager (75%) than the caregiver rating (56%). Inconsistency between both versions was low. Informativity increased from the 3L to the 5L version, nearly equally in both proxy ratings. The 5L also demonstrated a better discriminative ability and convergent validity than the 3L, especially in the caregiver rating.

**Conclusion:**

Compared to the EQ-5D-3L, the EQ-5D-5L had higher feasibility and acceptability and was slightly superior by a reduction of ceiling effects and an improvement in informativity, discriminative ability and convergent validity. Proxy ratings by informal caregivers overall demonstrated better psychometric properties than professional care-manager ratings.

**Supplementary Information:**

The online version contains supplementary material available at 10.1186/s12955-022-02049-y.

## Background

An essential goal of primary dementia care and psychosocial interventions for people living with dementia (PwD) is to improve health-related quality of life (HRQoL) [1–4]. However, the assessment of HRQoL among PwD is challenging due to the decline in cognitive capacity [5–8] and the limited perception of time, attention, judgment, and communication. These factors could affect the understanding and the completion of HRQoL questionnaires [6, 8, 9].

According to acceptability and validity, the preference-based health utility questionnaire EQ-5D performed comparably to other well-validated dementia-specific measures, e.g. the Quality of Life in Alzheimer's Diseases (QoL-AD) [10, 11]. This was underlined by two systematic reviews, concluding that the EQ-5D is a valid utility-based instrument for PwD and, therefore, recommended in use to measure HRQoL in this patient population [12, 13]. However, as dementia severity progresses, the collection of proxy ratings given by family members and informal caregivers or by medical and care staff instead of self-ratings was found to be more feasible [14].

Nevertheless, proxy-ratings demonstrate external perspectives of patients' HRQoL and should, hence, be used with caution [1, 15, 16]. Bryan et al. [17] found that data provided by clinicians and medical care staff had a higher construct validity compared to proxy ratings by informal caregivers for the more observable dimensions of HRQoL, e.g. patients' mobility and self-care. Conversely, caregiver ratings had a higher construct validity for the less observable dimension of HRQoL, e.g. depression and anxiety. Furthermore, there are also some differences, i.e. within proxy raters. For example, spouses rated HRQoL more positively than adult children [15]. These differences could significantly affect the conclusions drawn from HRQoL assessments.

Methodological drawbacks of the former version of the EQ-5D, the EQ-5D-3L, [18] prompted the development of a new five-level version, the EQ-5D-5L. This new instrument expands the range of the domains from three to five levels, aiming to improve discriminative ability, sensitivity, and responsiveness and reduce the ceiling effects [19, 20]. The psychometric properties of the three-level version compared to the five-level version have already been assessed in the general population and several chronic diseases. As a result, there are a marginal superiority of the five-level version in terms of increased informativity, discriminative ability, and decreased ceiling effects [21, 22].

However, no head-to-head comparison of the psychometric properties between the EQ-5D-3L and EQ-5D-5L in dementia diseases has been published, especially not comparing the commonly used proxy ratings. It is unknown whether an expansion from three to five levels in each dimension improves the deviation of proxy ratings by informal caregivers or health care professionals. Thus, this study aimed to compare the psychometric properties of the EQ-5D-5L with those of the 3L classification system in cognitively impaired PwD with patients' proxy ratings by family caregivers and care manager, both with a close patient-relationship.

## Methods

### Overview

The EQ-5D-3L, the EQ-5D-5L, the EQ-visual analogue scale (VAS) and the Quality of Life in Alzheimer's Diseases (QoL-AD) were completed as proxy ratings by family caregivers and care manager. Both versions of the EQ-5D were compared in terms of acceptability, agreement, ceiling effects, redistribution properties, inconsistency, informativity, and convergent and discriminative validity.

### Study design and recruitment

This study used data collected from the ongoing interventional study DCM:IMPact (Dementia Care Management: Implementation into different Care Settings), an implementation study, which builds on the DelpHi-trial (Dementia: Life- and person-centred help in Mecklenburg-Western Pomerania, Germany) [23]. The mixed-methods, multi-center, implementation study DCM:IMPact was initiated to evaluate the effectiveness and efficiency of collaborative dementia care [23, 24]. Effective [25] and cost-effective [26] dementia care management intervention was implemented in various care settings (e.g. physician networks, nursing care centers) to disclose which care setting would reveal the highest need and lowest implementation barriers for such models of care and, thus, where the best effects could be achieved.

Health care professionals assessed patients' eligibility (70 years or older, living at home, screened positive for dementia or received a formal dementia ICD-10 diagnosis). If patients were eligible, the professionals provided written and oral information about the study and asked for patient and caregiver written informed consent (IC). This study was approved by the local ethics committee at the University Medicine Greifswald (BB 01/2019).

This analysis was based on preliminary data, including n = 77 patients, n = 52 caregivers and one dementia care manager, who had provided collaborative dementia care management for six months. Data were assessed at baseline and three and six months after the baseline assessment.

### Data assessment

The EQ-5D-3L, the EQ-5D-5L, the EQ-VAS [18, 19, 27, 28], and the QoL-AD [29] were administered as proxy ratings via standardized computer-assisted face-to-face interviews. Thus, caregivers completed the measures via interview administrations at the caregivers' home done by a specifically-qualified nurse, the care manager. The care manager subsequently self-completed the EQ-5D-3L and 5L.

The informal caregivers and the care manager first completed the EQ-5D-3L with the EQ-VAS, followed by the completion of the EQ-5D-5L and the QoL-AD. Thus, for the caregiver, the "Interviewer Administered Proxy version 1" were used where the interviewer asked the caregiver (proxy) to rate the patient's health-related quality of life in their (the proxy's) opinion. For the care manager, we used the "Proxy version 1", where the care manager (the proxy) was asked to rate the patient's health-related quality of life in their (the proxy's) opinion. Interviews of the caregivers conducted by care manager were done first before the care manager themself completed the EQ-5D-3L and 5L.

### Health-related quality of life and clinical instruments

The EQ-5D is a generic HRQoL instrument containing three (no, some, and extreme problems) or five levels (no, slight, moderate, severe, and extreme problems) for the following five dimensions: mobility, self-care, pain/discomfort, usual activities, and anxiety/depression. The responses to the EQ-5D-3L were converted to health utilities, the preference-based single index measure for HRQoL anchored at 0 for death and 1 for full health [18, 19, 27, 28]. The QoL-AD is a dementia-specific HRQoL instrument consisting of 13 items (eg, physical health, living situation, family, mood, energy, cognition, relationships, activities, etc.) using a scale of 1–4 (poor, fair, good, or excellent). Results of the QoL-AD are presented as a sum score, ranging from 13 to 52. Higher scores indicate better quality of life [29].

The following sociodemographic and clinical data were assessed: cognitive impairment measured with the Mini-Mental State Examination (MMSE) [30], comorbidity assessed with the number of ICD-10 (International Statistical Classification of Diseases and Related Health Problems) diagnoses listed in the GP files [31] and the response to the Charlson Comorbidity Index [32], social functioning [33] and depression based on the Geriatric Depression Scale (GDS) [34], deficits in daily living activities based on the Bayer Activities of Daily Living (B-ADL) Scale [35], healthcare resource utilization, e.g. hospitalization, by application of the Resource Utilization in Dementia Questionnaire (RUD) [36], general mental and physical health (the dementia care manager subjectively categorized the patients' general health after completion of the intervention into one of the categories: very good, good, poor), and severity of pain assessed with the standardized assessment of older adults in primary care (STEP) [37].

### Data analyses

The responses to the EQ-5D-3L and EQ-5D-5L were converted to health utilities with the European and German value set [28, 38], respectively. The European value set of preference weights scores were applied to generate a VAS-based weighted health status index for all the potential 243 EQ-5D health states, ranging from 1 to − 0.074. The German value set is based on time trade-off and discrete choice experiments to estimate values for all 3125 possible health states, ranging from − 0.661 to 1. Descriptive statistics were used to present sociodemographic and clinical data for the study population. Measurement properties of the EQ-5D-3L and EQ-5D-5L were assessed in terms of acceptability, ceiling effects, agreement, redistribution properties, inconsistency, informativity, discriminative ability, and convergent validity.

#### Missing values and floor/ceiling effects

The number of missing values, the score ranges (observed vs. possible range), and the floor (% with lowest possible score) and ceiling effects (% with highest possible score) were used to compare the acceptability of both instruments. Additionally, absolute and relative changes in the ceiling effect of EQ-5D-3L versus EQ-5D-5L were calculated.

#### Agreement

The agreement between both versions was assessed with intraclass correlations (ICC) and presented with Bland–Altman plots. The ICC represents the proportion of variance from both index scores attributable to differences between individuals instead of the differences between the EQ-5D-3L and 5L. The higher the ICC, the higher agreement between the two versions. ICC higher (lower) than 0.7 indicates an acceptable (poor) agreement.

#### Redistribution properties and inconsistency

Inconsistency was assessed as suggested by previous studies [20, 39, 40], which defined a response within one EQ-5D domain as inconsistent when an answer in the three-level version is at least deviated two levels from the answer given in the five-level version (for example, 12,111 in the 3L version and 14,111 in the 5L version). The inconsistency size could thus range from 1 (two-level difference) to 3 (four-level difference). Redistribution properties were calculated as the percentage of consistent and inconsistent 3L–5L response pairs and the average size of inconsistency for each dimension and displayed with cross-tabulation of dimension scores.

#### Informativity

The informativity of both measures was assessed with Shannon indices (i.e. Shannon–Weaver index (H') and Shannon's evenness index (J')), which are appropriate measures to determine the discriminatory ability in health state classification in the comparison of the EQ-5D-3L and EQ-5D-5L. The higher the Shannon H' index, the more absolute information is captured by the measures. The Shannon Evenness index (J') captures the relative informativity of the distribution measure, regardless of the number of categories [20, 41]. If a cognitively impaired patient would not complete the additional levels as part of the 5L, relative informativity would be decreased, i.e. an expression of a loss of potential informativity [20, 42]. Discriminative power (change in absolute and relative informativity) was estimated for each dimension and the overall classification system. Positive (negative) values will demonstrate a gain (loss) of absolute and potential informativity of the 5L compared to the 3L version.

#### Known groups validity

The discriminative ability, defined as the ability to distinguish between different health and diseases stages, was assessed by different stages of functional impairment (Bayer Activities of daily living), cognitive impairment (Mini-Mental State Examination), depression (Geriatric depression scale) as well as general physical and mental health status. Cut-off values used for this analysis were established and validated within the development of each measure. Linear trends were assessed with the nonparametric Jonckheere trend test (> 3 groups) or Mann–Whitney test (2 groups).

#### Convergent validity

Convergent validity was assessed with Spearman's Correlation Coefficient between the EQ-5D-3L and EQ-5D-5L with the QoL-AD. Due to some overlap of dimensions (i.e. physical health, usual activities, self-care), we assumed there should be a moderate correlation between these measures. A correlation coefficient higher than 0.3 and 0.5 was determined as a moderate and strong correlation, respectively [43]. There should be a positive (negative) correlation between EQ-5D dimensions and B-ADL and GDS (EQ-VAS and QoL-AD) scores, as well as positive (negative) correlations between EQ-5D utility index and B-ADL and GDS (EQ-VAS and QoL-AD) scores.

## Results

### Patients' characteristics and health utility

The sample characteristics are presented in Table 1.

**Table 1:** Patients’ characteristics (n = 77)

*Sociodemographic and clinical characteristics*	
*Age*	
Mean (SD)	80.2 (6.4)
*Sex, n (%)*	
Female	47 (60.3)
*Living situation*	
Alone	27 (35.1)
*Functional impairment (B-ADL)*	
Mean, (SD)	3.3 (1.7)
No problems, n (%)	19 (29.2)
Moderate problems, n (%)	34 (52.3)
Severe problems, n (%)	12 (18.5)
*Cognitive impairment (MMSE)*	
Mean, (SD)	18.6 (7.4)
Mild, n (%)	43 (55.8)
Moderate to severe, n (%)	34 (44.2)
*Severity of pain, n (%)*	
No	26 (34.7)
Moderate	22 (29.3)
Severe	27 (36.0)
*General physical health status, n (%)*	
Very good	8 (10.4)
Good	43 (55.8)
Poor	26 (33.8)
*GDS*	
Mean (SD)	3.5 (3.2)
Depression, n (%)	17 (22.1%)

*B-ADL* bayer-activities of daily living scale, *MMSE* mini-mental state examination, *GDS* geriatric depression scale, *SD* standard deviation

There were 106 EQ-5D-3L and 5L proxy ratings by family caregivers (52 baseline, 15 three-months and 39 six-month follow-up assessments), and 133 EQ-5D-3L and 5L proxy ratings by the care manager (72 baselines, 20 three-months and 41 six-months follow-up assessments) were included in the analyses. The mean proxy-rating score of caregivers was 0.48 (SD 0.26) and 0.50 (SD 0.32) for the 3L and 5L, respectively. The mean health status caregivers stated in the VAS was 50.0 (SD 19.7). The care manager reported a higher mean utility score of 0.52 (SD 0.22) and 0.61 (SD 0.25) for the EQ-5D-3L and 5L. The mean value in the VAS by the care manager was 49.0 (SD 18.9), slightly lower as compared to the caregiver rating. The density plots and histograms of both measures and proxy ratings are demonstrated in Additional file 1: Figs. S1 and S3.

### Missing values

Missing values occurred comparably and less frequently in both versions (3L vs 5L). There was also a similar occurrence of missing values within proxy ratings (0.9% for caregiver ratings and 0.8% for care manager ratings), as demonstrated in Table 2.

**Table 2:** Missing values and ceiling effects

EQ-5D domains and utility value	Missing values		Ceiling effect		Ceiling effect reduction	
	3L (n, %)	5L (n, %)	3L (n, %)	5L (n, %)	Absolute (%) 3L%-5L%	Relative (%) Red n/3L n
*Proxy-rating (caregiver)*						
Mobility	1 (0.9)	1 (0.9)	26 (24.5)	16 (15.1)	9.4	38.5
Self-care	1 (0.9)	1 (0.9)	37 (34.9)	29 (27.4)	7.5	21.6
Usual activities	1 (0.9)	1 (0.9)	28 (26.4)	17 (16.0)	10.4	39.2
Pain/discomfort	1 (0.9)	1 (0.9)	40 (37.7)	33 (31.1)	6.6	17.5
Anxiety/depression	1 (0.9)	1 (0.9)	60 (56.6)	50 (47.2)	9.4	16.6
Overall (utility)	1 (0.9)	1 (0.9)	9 (8.5)	4 (3.8)	4.7	55.5
*Proxy-rating (care manager)*						
Mobility	10 (0.7)	11 (0.8)	33 (25.0)	16 (12.1)	12.9	51.5
Self-care	11 (0.8)	11 (0.8)	41 (30.8)	24 (18.1)	12.7	41.5
Usual activities	11 (0.8)	11 (0.8)	11 (8.3)	4 (3.0)	5.3	63.6
Pain/discomfort	11 (0.8)	11 (0.8)	85 (63.9)	58 (43.6)	20.3	31.8
Anxiety/depression	11 (0.8)	11 (0.8)	72 (54.6)	55 (41.4)	13.2	23.6
Overall (utility)	11 (0.8)	11 (0.8)	4 (3.0)	1 (0.8)	2.2	75.0

### Ceiling effects

Ceiling effects were for all domains smaller for the 5L compared to the 3L. The relative ceiling effect of the index decreased by 56% and 75% in the caregiver proxy rating and the case manager proxy rating, respectively. Ceiling effects were highest for the dimension "pain/discomfort" and "anxiety/depression". However, ceiling effects (8.5% vs. 3.0%) were higher and ceiling effect reduction lower (55.5% vs. 75.0%) in caregiver ratings than care manager ratings. Ceiling effects are depicted in Table 2.

### Agreement

The agreement between both versions was good, but slightly higher among caregivers (ICC = 0.885, CI 0.831–0.922; *p* < 0.001) than in care manager ratings (ICC = 0.840, CI 0.684–0.908; *p* < 0.001). The density plot, presented in Additional file 1: Fig. S1, shows that the EQ-5D-5L index scores were higher than the 3L's. The Bland–Altman Plots, presented in Additional file 1: Fig. S2, showed a mean difference between the 5L and 3L index values (5L–3L) of 0.02 (SD 0.16) and 0.08 (SD 0.19) for the caregiver and care manager rating, respectively. 91% of the mean differences in the caregiver-rating and 95% of the mean differences in the care manager-rating were within the confidence intervals. Fewer outlier differences were distributed above, most below the 95% confidence interval.

### Redistribution properties and inconsistency

The overall inconsistency of the EQ-5D proxy ratings was very low but slightly higher in caregiver ratings (caregiver: 2.6%, average size 1.09; care manager: 2.0%, average size 1.06). At least one inconsistent pair occurred in 14 (13%) out of 106 caregiver proxy assessments and 12 (9%) out of 133 care manager proxy assessments. The highest inconsistency was found for the dimension "pain/discomfort", with an average inconsistency size of 1.17 and 1.09, respectively. The redistribution properties and level of inconsistency are demonstrated in Table 3.

**Table 3:**
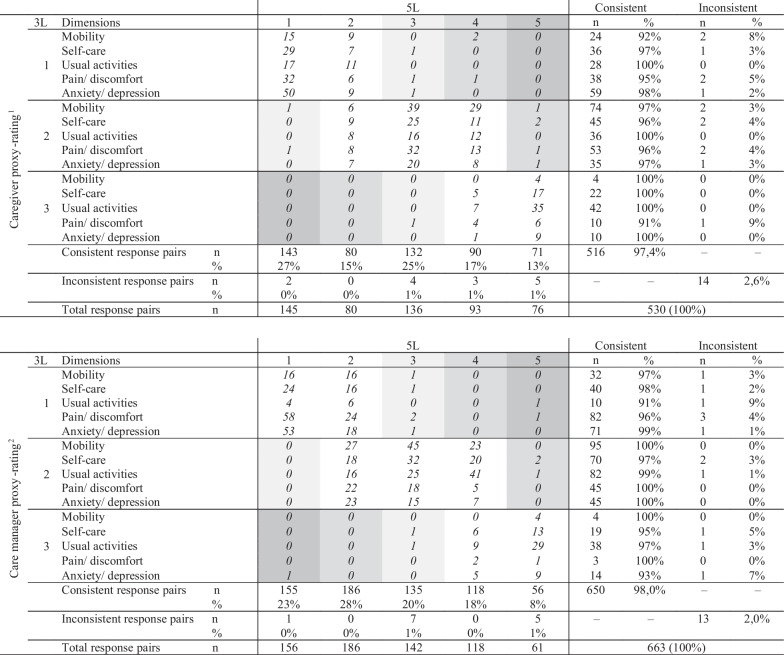
Redistribution properties from 3 to 5L responses and number of consistent and inconsistent respond pairs: a cross tabulation of dimension scores

^1^Redistribution properties from 3 to 5L responses of n = 106 EQ-5D-3L and 5L assessments, generating 530 response pairs 14 inconsistent pairs arise out of 14 of 106 assessments (13%). 14 inconsistent pairs arise out of 14 of 106 assessments (13%).

^2^Redistribution properties from 3 to 5L responses of n = 133 EQ-5D-3L and 5L assessments, generating 665 response pairs. 13 inconsistent pairs arise out of 12 of 133 assessments (9%); The size of inconsistency is represented in grayscale with more inconsistency in darker fields [51]

### Informativity

Absolute and relative informativity increased in the 5L compared to the 3L for both proxies, which demonstrated an average increase of the absolute (caregiver rating: + 0.56, care manager rating: + 0.51) and relative informativity (caregiver rating: + 0.06, care manager rating: + 0.10). The relative and absolute informativity increase was highest for the domain "mobility". Absolute and relative informativity are presented in Table 4.

**Table 4:** Inconsistency between the 3L and 5L and Shannon (H') and Shannon Evenness index (J')

	Assessments	Inconsistency		Shannon values 3L		Shannon values 5L	
	(n)	Inconsistency response pairs (n, %)	Average size of inconsistency (n, %)	H’	J’	H’	J’
*Caregiver proxy-rating*							
Mobility	106	4 (3.8%)	1.13	0.71	0.64	1.43	0.89
Self-care	106	3 (2.8%)	1.09	1.06	0.95	1.57	0.98
Usual activities	106	0 (0%)	1.00	1.08	0.98	1.56	0.96
Pain/discomfort	106	5 (4.7%)	1.17	0.94	0.85	1.56	0.96
Anxiety/depression	106	2 (1.9%)	1.07	0.91	0.82	1.39	0.87
Total/mean	–	14 (2.6%)	1.09	0.94	0.85	1.50	0.91
*Care manager proxy-rating*							
Mobility	133	1 (0.8%)	1.03	0.69	0.63	1.39	0.86
Self-care	133	4 (3.0%)	1.06	0.98	0.89	1.57	0.97
Usual activities	133	3 (2.3%)	1.07	0.86	0.78	1.42	0.89
Pain/discomfort	133	3 (2.3%)	1.09	0.73	0.67	1.23	0.76
Anxiety/depression	133	2 (1.5%)	1.07	0.94	0.86	1.38	0.86
Total/mean	–	13 (2.0%)	1.06	0.89	0.77	1.40	0.868

### Known groups validity

The EQ-5D-3L and EQ-5D-5L were equally able to discriminate between general physical and mental health stages, functional impairment, and patient hospitalizations. The five-level version of the EQ-5D better distinguishes between stages of depression and pain, demonstrating the superiority of the 5L over the 3L for caregiver ratings. Contrary to this, the EQ-5D-3L care manager rating better discriminates between stages of general physical and mental health, functional and cognitive impairment, and pain than the 5L. The discriminative ability is represented in Table 5.

**Table 5:** Discriminative ability/known-groups validity of the EQ-5D-3L and the EQ-5D-5L (proxy-rating given by a caregivers and care manager)

	Caregiver proxy rating				Care manager proxy rating			
	3L		5L		3L		5L	
	Mean (SD)	*p-value*	Mean (SD)	*p-value*	Mean (SD)	*p-value*	Mean (SD)	*p-value*
*Overall*								
Index values	0.48 (0.26)		0.50 (0.32)		0.52 (0.22)		0.61 (0.25)	
Visual Analogue Scale	0.50 (19.7)		0.50 (19.7)		0.49 (18.9)		0.49 (18.9)	
*MMSE*								
No hint for	0.63 (0.27)	0.114	0.60 (0.37)	0.272	0.58 (0.23)	**0.045**	0.71 (0.13)	0.073
Mild	0.49 (0.25)		0.55 (0.33)		0.56 (0.23)		0.65 (0.26)	
Moderate/Severe	0.44 (0.24)		0.45 (0.32)		0.47 (0.20)		0.55 (0.26)	
*General physical health*								
Very good	0.77 (0.31)	**0.001**	0.80 (0.23)	**0.001**	0.66 (0.24)	**0.007**	0.71 (0.31)	**0.044**
Good	0.49 (0.21)		0.53 (0.28)		0.53 (0.20)		0.63 (0.24)	
Poor	0.34 (0.18)		0.53 (0.32)		0.45 (0.21)		0.54 (0.26)	
*General mental health*								
Very good	0.68 (0.28)	**0.001**	0.73 (0.24)	**0.001**	0.57 (0.25)	**0.047**	0.67 (0.25)	0.102
Good	0.46 (0.26)		0.46 (0.38)		0.54 (0.20)		0.63 (0.23)	
Poor	0.38 (0.15)		0.42 (0.23)		0.45 (0.20)		0.54 (0.28)	
*Depression (GDS)*								
No hint for	0.50 (0.27)	0.066	0.54 (0.33)	**0.017**	0.53 (0.22)	0.322	0.62 (0.26)	0.213
Hint for	0.37 (0.17)		0.33 (0.33)		0.48 (0.22)		0.55 (0.26)	
*Functional impairment (B-ADL)*								
No problems	0.67 (0.27)	**0.001**	0.73 (0.24)	**0.001**	0.61 (0.21)	**0.011**	0.66 (0.26)	0.316
Moderate problems	0.43 (0.24)		0.48 (0.32)		0.49 (0.22)		0.59 (0.27)	
Severe problems	0.40 (0.20)		0.36 (0.27)		0.46 (0.19)		0.57 (0.23)	
*Pain (STEP)*								
No	0.53 (0.29)	**0.036**	0.61 (0.29)	**0.009**	0.47 (0.23)	**0.037**	0.56 (0.29)	0.057
Moderate	0.50 (0.27)		0.49 (0.41)		0.59 (0.20)		0.69 (0.16)	
Severe	0.39 (0.17)		0.39 (0.27)		0.50 (0.21)		0.58 (0.27)	
*Hospitalization*								
Yes	0.38 (0.19)	**0.001**	0.37 (0.32)	**0.001**	0.50 (0.21)	0.378	0.65 (0.25)	0.096
No	0.56 (0.27)		0.61 (0.29)		0.54 (0.22)		0.63 (0.32)	

Significant discriminations are highlighted in bold

### Convergent validity

Both proxy ratings demonstrated that the EQ-5D-5L had a better convergent validity with most of the measures, which revealed the superiority of the 5L version. However, the convergent validity was better for caregiver ratings than care manager ratings, demonstrated by larger correlation coefficients. The convergent validity of both measures is presented in Table 6.

**Table 6:** Convergent validity of the EQ-5D-3L and the EQ-5D-5L assessed using Spearman Correlation

	EQ-VAS (proxy-rating)		Qol-AD (patient self-rating)		Qol-AD (caregiver proxy-rating)		B-ADL (patient rating)		GDS (patient rating)	
	3L	5L	3L	5L	3L	5L	3L	5L	3L	5L
*Proxy (caregiver)*										
Mobility	‡	− **0.340**	− **0.438**	− 0.392	‡	‡	‡	‡	‡	‡
Self-care	− 0.331	− **0.392**	‡	− **0.365**	− 0.519	− **0.613**	‡	‡	‡	**0.320**
Usual activities	− 0.459	− **0.482**	‡	− **0.302**	− 0.542	− **0.552**	**0.325**	0.305	0.340	**0.366**
Pain/discomfort	‡	‡	− 0.303	− **0.307**	‡	‡	‡	‡	‡	‡
Anxiety/depression	‡	‡	‡	‡	‡	‡	‡	‡	‡	‡
EQ-5D index score	0.454	**0.471**	0.399	**0.457**	**0.486**	0.444	‡	‡	‡	− **0.306**
*Proxy (care manager)*										
Mobility	− 0.479	− **0.639**	− **0.381**	− 0.326	‡	‡	‡	‡	‡	‡
Self-care	− 0.692	− **0.720**	‡	− **0.360**	− 0.411	− **0.478**	‡	‡	‡	‡
Usual activities	− 0.660	− **0.722**	‡	‡	‡	− **0.373**	‡	**0.300**	‡	**0.305**
Pain/discomfort	‡	‡	‡	‡	‡	‡	‡	‡	‡	‡
Anxiety/depression	‡	‡	‡	‡	‡	‡	‡	‡	‡	‡
EQ-5D index score	**0.698**	0.643	0.312	**0.326**	**0.412**	0.341	‡	‡	− 0.324	− **0.350**

*SD* standard deviation, ‡ poor correlation represents values less than 0.3, *QoL-AD* Quality of Life in Alzheimer's Diseases, *ADL* Activities of Daily Living, *GDS* Geriatric Depression Scale, superior correlations are demonstrated in bold

## Discussion

To the best of our knowledge, this is the first analysis comparing the psychometric properties of the EQ-5D-5L compared to the EQ-5D-3L in PwD using proxy ratings given by informal family caregivers and health professionals. Generally, the EQ-5D-5L reveals higher index scores than the 3L. Both EQ-5D-5L proxy ratings improve psychometric properties by reducing ceiling effects and improving informativity and convergent validity. As demonstrated by the ICC, the agreement between the three- and five-level EQ-5D was excellent but slightly higher in the caregiver proxy rating than the care manager rating. Also, caregiver proxy ratings demonstrated a better convergent validity than the care manager proxy ratings. Thus, the EQ-5D-5L shows its superiority over the 3L version as a proxy rating used in dementia, primarily when family caregivers assess patients' health status.

Both EQ-5D-5L proxy measures demonstrated similar feasibility, acceptability (missing values), informativity, and consistency. The agreement between both proxy-rating measures was excellent (ICC 0.885 for caregiver rating and 0.840 for care manager rating) and in line with the reported agreement in previous studies, revealing ICC higher than 0.85 [44, 45]. Missing values infrequently occurred, comparable to previously published studies [20, 44, 46].

Several validation studies found ceiling effects of both measures in different settings between 15 and 50%, with a decrease of up to 10% from the 3L to the 5L version [20, 44, 45, 47–50]. The ceiling effects reported by proxies in this analysis were lower (< 8.5%) than in these previous studies, underlining the excellent feasibility of both EQ-5D-5L proxy ratings in dementia. Even though the proportion of patients in "full health" was lower, the decreased absolute and relative ceiling effect of the EQ-5D-5L was in line with other studies' findings [20, 44–50].

In line with previously published studies, this analysis demonstrated a significant gain in absolute informativity and an improvement in relative informativity for all dimensions in the EQ-5D-5L [20, 44, 45, 51]. The underlying sample of older and, in most cases, multimorbid PwD could be the main reason for choosing all response levels in the 5L to describe patients' health by family caregivers or the care manager, which causes a higher variation of health states. This is in line with previously published studies [20, 52].

The extension from three to five levels could be more challenging, causing inconsistent valuations. However, informal caregiver and care manager proxy ratings revealed a very low inconsistency (< 3%), in line with previous studies (1–5%) [44, 45, 48, 53]. Thompson et al. underlined that inconsistency is higher in populations with multimorbidity (up to 10%) than in the general population (4%). Still, inconsistency appeared to be low in caregiver and care manager proxy ratings, as demonstrated in this analysis. This could mean that proxies, e.g. caregivers or staff, can reliably assess patients' health status.

A systematic review by Hounsome et al. [54] summarized different aspects of the performance of the EQ-5D in studies of dementia, which revealed that other proxies, e.g. family carers and health care professionals, provide separate ratings for patients' health. The authors further concluded that the mode of assessment and selecting appropriate proxies is vital to ensure a high validity in this specific sample. Further studies report that both instruments, the EQ-5D-3L and 5L, demonstrate a good known-groups validity in dementia diseases, with some evidence that the 5L discriminates better between different groups [44–46], which caregiver ratings in this analysis could only confirm. For the care manager, the EQ-5D-3L distinguished better between stages of health, suggesting that we could not demonstrate an overall superiority of the EQ-5D-5L over the EQ-5D-3L in the known-groups validity. Also, caregiver ratings discriminate better between health states than ratings of health professionals, demonstrating the superiority of caregiver ratings over ratings of health professionals.

Both proxy-rating instruments also performed well regarding the convergent validity, with evidence tendency for a slightly better convergent performance of the EQ-5D-5L. However, this superiority remains uncertain and should be confirmed in future psychometric head-to-head analyses that compare both measures in dementia diseases. Most previously published studies reported a slight to considerate superiority of the EQ-5D-5L concerning the convergent validity [44, 45]. However, the caregiver rating's convergent validity was slightly higher than the care manager rating, which demonstrates that the EQ-5D-5L administered in caregivers outperforms the care manager proxy rating. A study by Bryan et al. [17] sought to identify whether the validity of the EQ-5D was higher for family caregivers or health care professionals. Their findings suggest that EQ-5D ratings by family caregivers had a higher validity for less observable dimensions, i.e. "usual activities" and "anxiety and depression". In contrast, the construct validity in health care professional ratings was higher for the more observable dimensions of the EQ-5D, i.e. "mobility" and "self-care". This could, however, not be confirmed by our results.

### Strengths and limitations

This is the first study administrating both the three and five-level versions of the EQ-5D in multiple proxies, creating a sufficient basis for a comprehensive assessment of the psychometric performance of the EQ-5D in dementia. Furthermore, a strength of this analysis was the inclusion of several in this indication critical clinical measures, like cognitive and functional impairment, general health, depression and pain, which were needed to assess the validity of the EQ-5D instruments thoroughly.

However, several limiting aspects are acknowledged. First of all, the study was based on a sample size of 106 caregiver assessments and 131 care manager proxy ratings, limiting the generalizability of the results. Especially the fact that only n = 52 caregivers and only one heath professional (care manager) assessed patients' health limits the robustness of the presented results. Secondly, the EQ-5D-3L was completed before the EQ-5D-5L by caregivers and the health professional (care manager). Thus, overuse of levels two and four in the 5L could be possible, as reported by Janssen et al. [39]. Furthermore, the mode of administration differed between caregiver proxy-rating (interview) and care manager ratings (self-rating). This could further limit the generalizability of the presented results.

Contrary to this, an initial completion of the 5L could have caused the overuse of level two in the 3L version. Future studies should consider randomization of the application process to reduce potential bias. Most importantly, the care manager completed the EQ-5D-3L and 5L after interviewing the caregiver, who stated the patient's health as a proxy. Therefore, this survey sequence might have influenced the care manager in assessing the patient's health status.

Finally, the comparison of the psychometric performance is affected by the different value sets used. While the European value set used for the EQ-5D-3L caused a range of utility values between – 0.074 and 1, the German value set led to EQ-5D-5L utility values between − 0.661 to 1. Thus, the EQ-5D-5L basically had a wider range that could be an advantage in distinguishing groups of diseases stages and general health, and also to correlate with other measures. While the agreement between the measures (3L and 5L) was excellent and both measures performed equally in the known groups' validity, the 5L had a better convergent validity, which could also be due to the basic differences in the different value sets used. Further research is needed to reveal the impact on the psychometric properties revealed within head-to-head comparisons. Cross-walks that mapped responses of the EQ-5D-3L to the EQ-5D-5L could be helpful and a prerequisite to use the same value set for both measures.

## Conclusion

Our results provide some indications that the five-level version of the EQ-5D was slightly superior over the three-level version by improving informativity and convergent and discriminative validity and reducing ceiling effects. Our findings also indicate that family caregivers' ratings may be preferable to measure HRQoL in PwD due to a better discriminatory ability and higher convergent validity. However, further research is needed to clarify and confirm the superiority of the five-level version of the EQ-5D using larger sample size and also taking reliability and responsiveness measures into account.

## Supplementary Information

**Additional file 1: Figure S1**. Density plots of the self- and proxy-rating EQ-5D-3L and 5L index values. **Figure S2**. Bland-Altman plots of the EQ-5D-3L and the EQ-5D-5L index values Vertical Axis represents the difference between the EQ-5D-5L and EQ-5D-3L (5L minus 3L).**Figure S3**. Histograms of the self- and proxy-rating EQ-5D-3L and 5L index values.

## Data Availability

After request.

## Code availability

After request.

## References

[1] Romhild J (2018). Inter-rater agreement of the Quality of Life-Alzheimer's Disease (QoL-AD) self-rating and proxy rating scale: secondary analysis of RightTimePlaceCare data. Health Qual Life Outcomes.

[2] Dichter MN (2016). Linguistic validation and reliability properties are weak investigated of most dementia-specific quality of life measurements-a systematic review. J Clin Epidemiol.

[3] Ettema TP (2005). The concept of quality of life in dementia in the different stages of the disease. Int Psychogeriatr.

[4] Lawton MP (1997). Assessing quality of life in Alzheimer disease research. Alzheimer Dis Assoc Disord.

[5] Bowling A (2015). Quality of life in dementia: a systematically conducted narrative review of dementia-specific measurement scales. Aging Ment Health.

[6] Rabins PV, Black BS (2007). Measuring quality of life in dementia: purposes, goals, challenges and progress. Int Psychogeriatr.

[7] Whitehouse PJ, Rabins PV (1992). Quality of life and dementia. Alzheimer Dis Assoc Disord.

[8] Logsdon RG (2002). Assessing quality of life in older adults with cognitive impairment. Psychosom Med.

[9] Thorgrimsen L (2003). Whose quality of life is it anyway? The validity and reliability of the Quality of Life-Alzheimer's Disease (QoL-AD) scale. Alzheimer Dis Assoc Disord.

[10] Naglie G (2006). Utility-based Quality of Life measures in Alzheimer's disease. Qual Life Res.

[11] Silberfeld M (2002). Content validity for dementia of three generic preference based health related quality of life instruments. Qual Life Res.

[12] Li L (2018). Utility-based instruments for people with dementia: a systematic review and meta-regression analysis. Value Health.

[13] Yang F (2018). Measurement tools of resource use and quality of life in clinical trials for dementia or cognitive impairment interventions: a systematically conducted narrative review. Int J Geriatr Psychiatry.

[14] Martin A (2019). How should we capture health state utility in dementia? Comparisons of DEMQOL-proxy-U and of self- and proxy-completed EQ-5D-5L. Value Health.

[15] Orgeta V (2015). The use of the EQ-5D as a measure of health-related quality of life in people with dementia and their carers. Qual Life Res.

[16] Dichter MN (2018). Item distribution and inter-rater reliability of the German version of the quality of life in Alzheimer's disease scale (QoL-AD) proxy for people with dementia living in nursing homes. BMC Geriatr.

[17] Bryan S (2005). Proxy completion of EQ-5D in patients with dementia. Qual Life Res.

[18] Devlin NJ, Krabbe PF (2013). The development of new research methods for the valuation of EQ-5D-5L. Eur J Health Econ.

[19] Herdman M (2011). Development and preliminary testing of the new five-level version of EQ-5D (EQ-5D-5L). Qual Life Res.

[20] Janssen MF (2013). Measurement properties of the EQ-5D-5L compared to the EQ-5D-3L across eight patient groups: a multi-country study. Qual Life Res.

[21] Ferreira LN (2016). Comparing the performance of the EQ-5D-3L and the EQ-5D-5L in young Portuguese adults. Health Qual Life Outcomes.

[22] Bas Janssen MF, Birnie E, Bonsel GJ (2007). Evaluating the discriminatory power of EQ-5D, HUI2 and HUI3 in a US general population survey using Shannon's indices. Qual Life Res.

[23] Thyrian JR (2012). Life- and person-centred help in Mecklenburg-Western Pomerania, Germany (DelpHi): study protocol for a randomised controlled trial. Trials.

[24] Eichler T (2014). Dementia care management: going new ways in ambulant dementia care within a GP-based randomized controlled intervention trial. Int Psychogeriatr.

[25] Thyrian JR (2017). Effectiveness and safety of dementia care management in primary care: a randomized clinical trial. JAMA Psychiat.

[26] Michalowsky B (2019). Cost-effectiveness of a collaborative dementia care management-Results of a cluster-randomized controlled trial. Alzheimers Dement.

[27] Rabin R, de Charro F (2001). EQ-5D: a measure of health status from the EuroQol Group. Ann Med.

[28] Greiner W (2003). A single European currency for EQ-5D health states: Results from a six-country study. Eur J Health Econ.

[29] Hylla J (2016). Internal consistency and construct validity of the Quality of Life in Alzheimer's Disease (QoL-AD) proxy: a secondary data analysis. Pflege.

[30] Kessler, J., H.J. Markowitsch, and P. Denzler, *Mini-Mental-Status-Test (MMST) [German Version]*. 1990, Göttingen: Beltz Test GmbH.

[31] World Health Organization *The ICD-10 classification of mental and behavioural disorders: Diagnostic criteria for research*. http://www.who.int/classifications/icd/en/GRNBOOK.pdf, 1993.

[32] Charlson ME (1987). A new method of classifying prognostic comorbidity in longitudinal studies: development and validation. J Chronic Dis.

[33] Fydrich T, Sommer G, Brähler E (2007). F-SozU: Fragebogen zur sozialen Unterstützung.

[34] Gauggel S, Birkner B (1999). Validity and reliability of a German version of the Geriatric Depression Scale (GDS). Zeitschrift für Klinische Psychologie-Forschung und Praxis.

[35] Hindmarch I (1998). The Bayer Activities of Daily Living Scale (B-ADL). Dement Geriatr Cogn Disord.

[36] Wimo A, Nordberg G (2007). Validity and reliability of assessments of time: Comparisons of direct observations and estimates of time by the use of the resource utilization in dementia (RUD)-instrument. Arch Gerontol Geriatr.

[37] Sandholzer H (2004). STEP–standardized assessment of elderly people in primary care. Dtsch Med Wochenschr.

[38] Ludwig K, Graf von der Schulenburg JM, Greiner W (2018). German value set for the EQ-5D-5L. Pharmacoeconomics.

[39] Janssen MF (2008). Comparing the standard EQ-5D three-level system with a five-level version. Value Health.

[40] Pickard AS (2007). Psychometric comparison of the standard EQ-5D to a 5 level version in cancer patients. Med Care.

[41] Pickard AS (2007). Evaluating equivalency between response systems: application of the Rasch model to a 3-level and 5-level EQ-5D. Med Care.

[42] Scalone L (2013). Comparing the performance of the standard EQ-5D 3L with the new version EQ-5D 5L in patients with chronic hepatic diseases. Qual Life Res.

[43] Hinkle DW (2003). Applied statistics for the behavioral sciences.

[44] Yfantopoulos J, Chantzaras A, Kontodimas S (2017). Assessment of the psychometric properties of the EQ-5D-3L and EQ-5D-5L instruments in psoriasis. Arch Dermatol Res.

[45] Yfantopoulos JN, Chantzaras AE (2017). Validation and comparison of the psychometric properties of the EQ-5D-3L and EQ-5D-5L instruments in Greece. Eur J Health Econ.

[46] Rencz F (2019). Validity of the EQ-5D-5L and EQ-5D-3L in patients with Crohn's disease. Qual Life Res.

[47] Poor AK (2017). Measurement properties of the EQ-5D-5L compared to the EQ-5D-3L in psoriasis patients. Qual Life Res.

[48] Thompson AJ, Turner AJ (2020). A Comparison of the EQ-5D-3L and EQ-5D-5L. Pharmacoeconomics.

[49] Eneqvist T (2020). How do EQ-5D-3L and EQ-5D-5L compare in a Swedish total hip replacement population?. Acta Orthop.

[50] Greene ME (2015). The EQ-5D-5L improves on the EQ-5D-3L for health-related quality-of-life assessment in patients undergoing total hip arthroplasty. Clin Orthop Relat Res.

[51] Buchholz I (2018). A systematic review of studies comparing the measurement properties of the three-level and five-level versions of the EQ-5D. Pharmacoeconomics.

[52] Agborsangaya CB (2014). Comparing the EQ-5D 3L and 5L: measurement properties and association with chronic conditions and multimorbidity in the general population. Health Qual Life Outcomes.

[53] Kim TH (2013). Psychometric properties of the EQ-5D-5L in the general population of South Korea. Qual Life Res.

[54] Hounsome N, Orrell M, Edwards RT (2011). EQ-5D as a quality of life measure in people with dementia and their carers: evidence and key issues. Value Health.

