# Supplementary material for: Comparing the psychometric properties of EQ-5D-3L and EQ-5D-5L proxy ratings by informal caregivers and a health professional for people with dementia
Source: Health Qual Life Outcomes. 2022 Oct 5;20:140. doi: 10.1186/s12955-022-02049-y (PMC9535990; doi:10.1186/s12955-022-02049-y)
Supplement: Supplementary file 1 — Additional file 1: Figure S1. Density plots of the self- and proxy-rating EQ-5D-3L and 5L index values. Figure S2. Bland-Altman plots of the EQ-5D-3L and the EQ-5D-5L index values Vertical Axis represents the difference between the EQ-5D-5L and EQ-5D-3L (5L minus 3L).Figure S3. Histograms of the self- and proxy-rating EQ-5D-3L and 5L index values. [file 12955_2022_2049_MOESM1_ESM.docx]

**
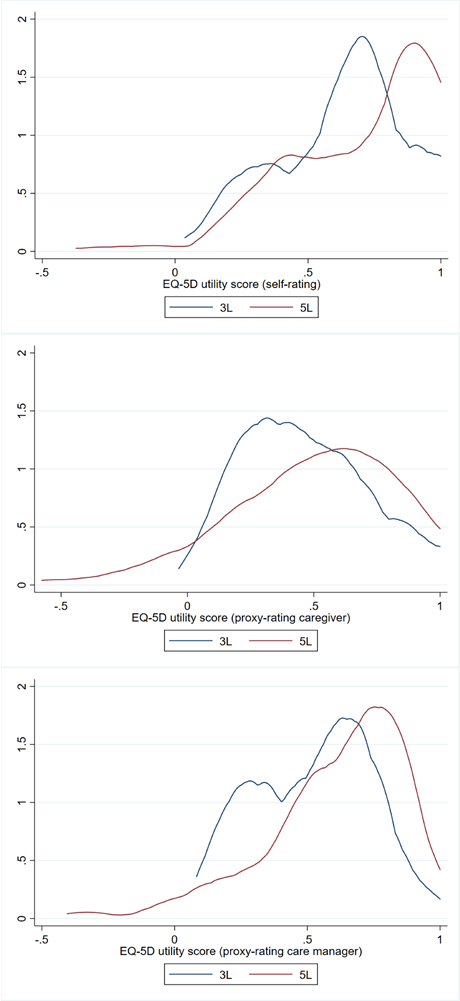
**

Supplementary Figure 1: Density plots of the self- and proxy-rating EQ-5D-3L and 5L index values


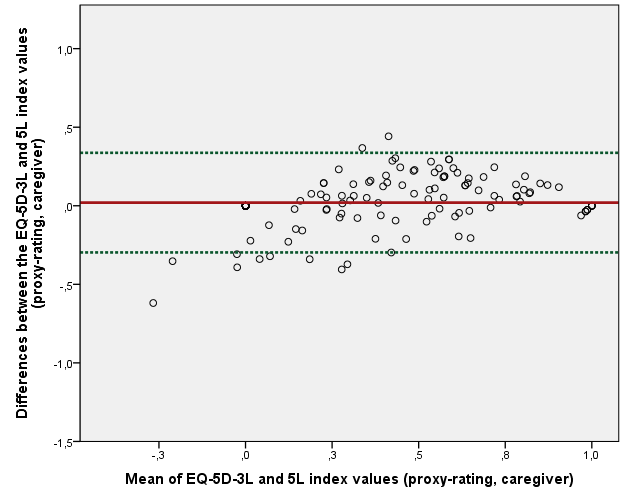


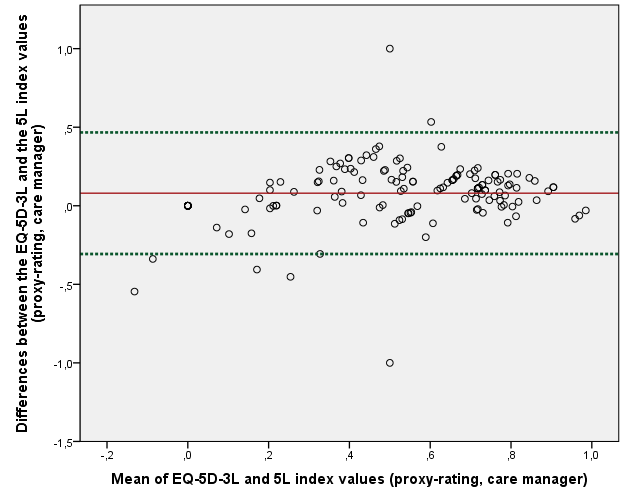


Supplementary Figure 2: Bland-Altman plots of the EQ-5D-3L and the EQ-5D-5L index values

*Vertical Axis represents the difference between the EQ-5D-5L and EQ-5D-3L (5L minus 3L)*


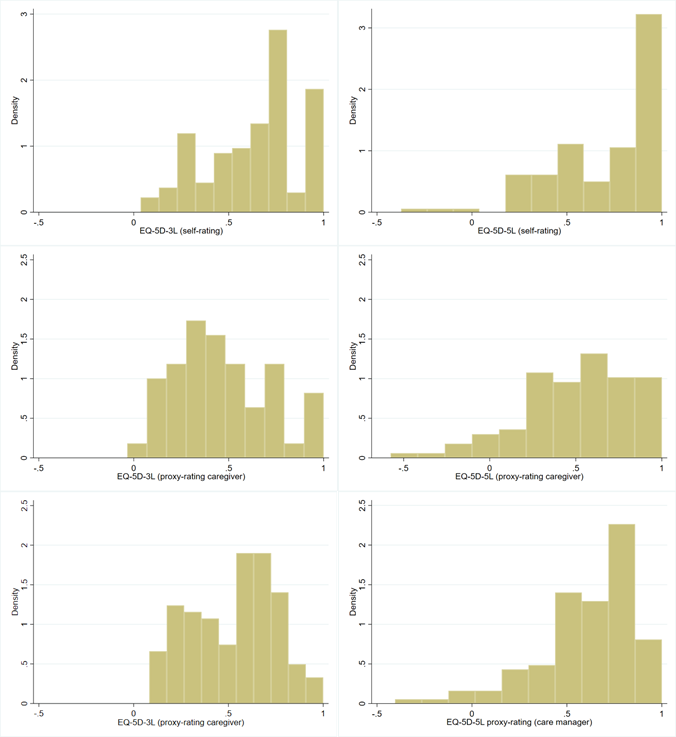


Supplementary Figure 3: Histograms of the self- and proxy-rating EQ-5D-3L and 5L index values
